# Safety of Long-Acting Intravitreal Hydrogels: A Comprehensive Evaluation of Intraocular Pressure and Retinal Integrity across Rabbit and Non-Human Primate Models

**DOI:** 10.1021/acsptsci.6c00243

**Published:** 2026-07-12

**Authors:** Alexa K. Beathard-Wojan, Becci Boughner, Vibhuti Agrahari

**Affiliations:** † Department of Pharmaceutical Sciences, University of Oklahoma Health Campus, 1110 N Stonewall Ave, Oklahoma City, Oklahoma 73117, United States; ‡ Oklahoma School of Science and Mathematics, 1141 N Lincoln Blvd, Oklahoma City, Oklahoma 73104, United States

**Keywords:** animal model, ocular drug delivery, posterior segment, nanotechnology, polymeric scaffolds

## Abstract

Injectable hydrogels have emerged as promising platforms for sustained drug delivery to the posterior segment of the eye, with the potential to reduce treatment burden and improve patient adherence in chronic disease management. Although numerous preclinical studies report favorable biocompatibility and biodegradability, the effects of intravitreal (IVT) hydrogels on the ocular environment have yet to be comprehensively evaluated. In this study, we conducted a meta-analysis of preclinical investigations evaluating IVT, drug-loaded hydrogels in rabbit and nonhuman primate models. Primary safety end points included longitudinal changes in intraocular pressure (IOP) and total retinal thickness (TRT). Across all included studies, no significant or sustained elevations in IOP were observed relative to baseline or untreated controls. TRT remained stable for 14 days up to two months postadministration, with no evidence of clinically meaningful thinning, degeneration, or retinal toxicity. These outcomes were consistent across diverse hydrogel compositions, therapeutic payloads, gelation mechanisms, and animal models, indicating a broadly favorable intraocular safety profile. Collectively, this analysis provides quantitative evidence supporting the ocular safety of IVT hydrogel-based drug delivery systems based on IOP and TRT measurements in preclinical models. These findings reinforce the translational potential of injectable hydrogels as biocompatible, long-acting alternatives to conventional IVT injection–based therapies and support their continued development for intraocular applications.

## Graphical abstract

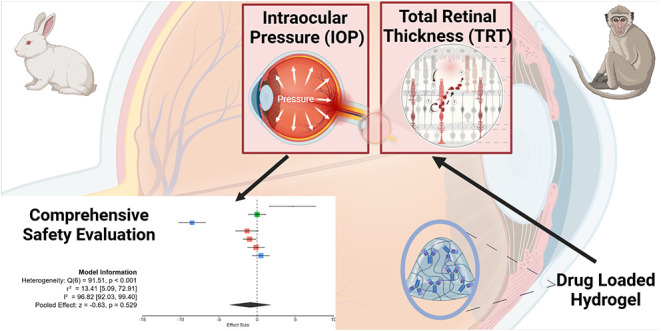

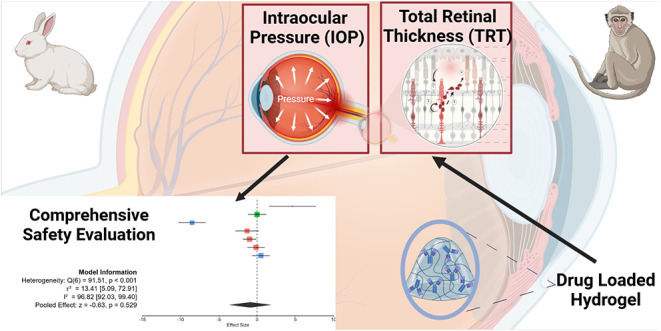

Intravitreal (IVT) injections have become one of the most commonly performed ophthalmic procedures, driven largely by the success of antivascular endothelial growth factor (anti-VEGF) agents and corticosteroids in treating retinal diseases such as neovascular age-related macular degeneration (nAMD), diabetic macular edema, retinopathy of prematurity, and retinal vein occlusion.^1,2^ The global burden of these diseases continues to rise, with over 20 million injections administered annually.^3^

Although IVT injections are generally considered safe, repeated administration exposes intraocular tissues to cumulative mechanical and biological stress. Reported complications include intraocular hemorrhage, retinal detachment, photoreceptor degeneration, cataract formation, and bacterial endophthalmitis.^4^ The most frequently observed immediate physiological response is a transient elevation in intraocular pressure (IOP) caused by acute volume displacement. While short-lived IOP spikes are typically tolerated in otherwise healthy eyes, some patients experience chronic IOP elevations after injection.^5^ Additionally, postinjection IOP fluctuations have been associated with thinning of the retinal nerve fiber layer, raising concerns regarding long-term optic nerve health.^6^ Beyond these physiological risks, the need for frequent injections imposes a significant burden on both patients and healthcare systems, particularly retina specialists tasked with meeting growing clinical demand.^3^

Given the chronic nature of retinal diseases, considerable effort has been directed toward developing sustained-release drug delivery systems that safely maintain therapeutic concentrations within the posterior segment of the eye. The SUSVIMO refillable implant, approved in 2021 for nAMD, provides continuous delivery of ranibizumab over approximately six months.^7–9^ However, its use requires surgical implantation, periodic refilling, and potential surgical removal, which elevate the risk of complications such as infection, conjunctival erosion, and inadequate wound closure.^7,10,11^ Biodegradable implants overcome issues related to refill and removal, as exemplified by the dexamethasone implant Ozurdex; yet these systems still carry risks including sustained IOP elevation, retinal detachment, inflammation, and infection.^12^ Meta-analytic evidence indicates that while IOP elevations following anti-VEGF injections typically resolve within days, corticosteroid implants such as Ozurdex may induce pressure increases lasting months, highlighting the limitations of current long-acting intraocular platforms.^13^

In this context, hydrogels have emerged as a promising alternative for long-acting drug delivery to the posterior segment. Hydrogels offer key advantages including biocompatibility, biodegradability, tunable mechanical properties, and the capacity for long-term drug release. Their viscoelastic nature allows them to withstand ocular fluid dynamics while minimizing tissue damage at the site of administration.^14,15^ High water content and favorable mesh architecture support delivery of sensitive biologics, such as proteins, peptides, nucleic acids, and even living cells, while protecting them from degradation and rapid clearance.^15^ For ocular applications, hydrogels are particularly attractive because their physical properties closely resemble the native vitreous humor, enabling enhanced compatibility and residence time. They can also be engineered to codeliver multiple agents and to undergo sol-to-gel transitions in response to environmental triggers, enabling minimally invasive administration of depot-forming systems.^16–18^ However, hydrogels differ fundamentally from small-volume solutions or implants. Once injected, they undergo swelling, gelation, degradation, and long-term residency within the vitreous cavity, each of which may affect IOP homeostasis, vitreous architecture, or retinal integrity. Thus, although hydrogels have the potential to substantially reduce the treatment burden, their introduction as long-lasting intraocular materials introduces new safety considerations that must be evaluated.

Despite numerous preclinical studies exploring hydrogel-based drug delivery for IVT applications,^19–21^ there remains no comprehensive synthesis of how intravitreally administered hydrogels, whether in situ gelling or preformed, affect key ocular safety parameters such as IOP and TRT. The present study aims to systematically evaluate the ocular safety profile of IVT hydrogels using data from preclinical rabbit and nonhuman primate (NHP) models. By positioning hydrogel performance within the established safety landscape of IVT injections, this study demonstrates that hydrogels maintain stable IOP and TRT for at least two months postadministration, supporting their safety and biocompatibility as intraocular drug-delivery scaffolds.

## 2 Results

### 2.1 Study Characteristics

Of the 359 unique studies returned in the initial search strategy (section 4.1), only nine studies met all inclusion criteria and were included in the final meta-analysis. Among the nine eligible studies, six employed rabbit models and three utilized nonhuman primates (NHPs) (Figure 1). Included studies were grouped according to assessed time points, availability of IOP and/or TRT measurements, and the type of control used to determine eligibility for each meta-analysis. Raw data used in the final analyses are provided in Tables S1 and S2.

**Figure 1:**
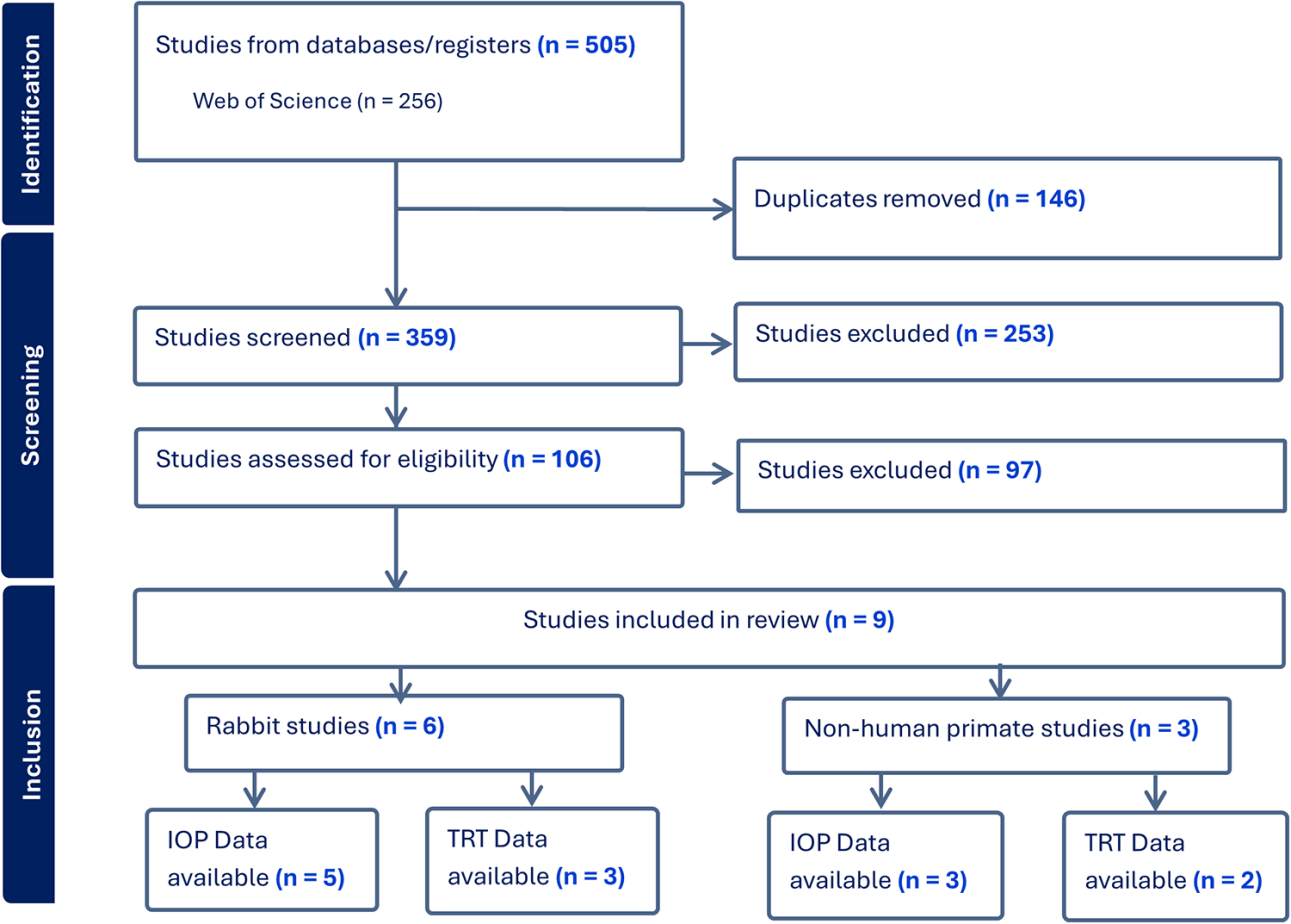
PRISMA diagram for study identification, screening, selection, exclusion, and retention at each stage.

Only one study (Duan et al., 2023)^22^ investigated a diseased model, while all remaining studies were conducted in healthy animals. Rabbit ages ranged from 2 to 6 months, whereas NHPs were between 5 and 10 years of age (Table 1). The primary therapeutic target across studies was retinal neovascularization, with hydrogel formulations encompassing a wide range of polymer compositions. Delivery strategies included injectable preformed gels (3/9), surgically implanted preformed gels (2/9), and in situ forming systems that gelled following IVT injection (4/9). The median injection volume was 50 μL, with reported volumes ranging up to 100 μL. Control conditions varied across studies and included no treatment, saline or PBS injection, free drug solution injection, and/or blank (drug-free) hydrogels (Table 2).

**Table 1:** Population Details for Included Studies

study ID	animal model used	diseased state (if applicable)	age of animals	weight of animals (if included)	gender of animals	control eyes on same animal or different.
Duan et al. (2023)^22^	chinchilla rabbits	rabbit persistent retinal neovascularization model	10–12 weeks	2.2–2.5 kg	male	different
Shen et al. (2023)^23^	new zealand white rabbits		8–12 weeks		male	different
Hu et al. (2024)^24^	new zealand white rabbits	healthy rabbits used for safety assessments (although diseased model used in other study aspects)	3–4 months	2.8 to 3.1 kg	female	different
Meany et al. (2023)^25^	new zealand white rabbits		6 months		male	different
da Silva et al. (2025)^26^	new zealand white rabbits		2–3 months	2.0–2.5 kg	male	same
Rauck et al. (2014)^27^	new zealand white rabbits		8 weeks		male	not specified
Story et al. (2025)^28^	rhesus macaques (*Macaca mulatta*)		6.8 ± 1.7 years (range 3–10 years)	10.0 ± 3.4 kg (male: 12.7 ± 3.1 kg, female: 7.8 ± 1.5 kg)	11 males and 12 females	same
Kim et al. (2020)^29^	rhesus macaques (*Macaca mulatta*)		7.6 ± 2.6 years	9.3 ± 3.3 kg	4 male, 6 female	same
Patel et al. (2022)^30^	cynomolgus monkeys		not available	not available	not available	same

**Table 2:** Treatment Details for Included Studies

study ID	disease targeted	drug used	hydrogel components	incision made prior to injection (Yes or no)	needle specifications	gelling mechanism	volume of gel injected	control(s) used
Duan et al. (2023)^22^	retinal neovascularization	ranibizumab	HA–ADH, PBS; PF127-CHO	no	not specified	preformed gel; injectable	100 μL	(1) no injections/implants; (2) drug injected, no hydrogel; (3) PBS/saline injection, no hydrogel; (4) hydrogel only, no drug; (5) baseline
Shen et al. (2023)^23^	posterior eye diseases	triamcinolone acetonide	GelMA; LAP	yes	1 mL syringe	in situ gel, light responsive	50 μL	(1) no injections/implants; (2) hydrogel only, no drug; (3) baseline
Hu et al. (2024)^24^	sodium-iodate-induced retinal degeneration	GDNF-secreting HEK cells	neutralized collagen type I; NaOH; alginate solution	yes, and small punctures made in eye to release IOP.	0.5 mm thick gel-loading pipet tips	preformed gel; injectable	30 or 60 μL	(1) no injections/implants; (2) PBS/saline injection, no hydrogel
Meany et al. (2023)^25^	glaucoma	bimatoprost	HPMC-C12; PEG–PLA NPs	no	0.3 mL insulin syringe, 31-gauge × 8 mm needle	preformed gel; injectable	50 μL	(1) hydrogel only, no drug; (2) baseline
da Silva et al. (2025)^26^	glaucoma	acetazolamide	PLGA	no	25-gauge trocar	implant	5.0 mm in length and 0.45 mm in thickness	(1) drug injected, no hydrogel; (2) implant only, no drug; (3) baseline
Rauck et al. (2014)^27^	neovascularization of the retina	Bevacizumab	PSHU	no	31-gauge needle	in situ gel, temperature responsive	50 μL	(1) no injections/implants; (2) drug injected, no hydrogel; (3) baseline
Story et al. (2025)^28^	age related macular degeneration (AMD)	aflibercept-loaded PLGA microspheres	PEG–PLLA-DA, NIPAAm, N-tert-butylacrylamide, TEMED, and ammonium persulfate	no	27-gauge hypodermic needle and tuberculin syringe	in situ gel, temperature responsive	50 μL	(1) no injections/implants; (2) baseline
Kim et al. (2020)^29^	retinal conditions	aflibercept-loaded PLGA microspheres	NIPAAm, N-tert-butylacrylamide, PEG-DA, TEMED, and ammonium persulfate.	no	22-gauge needle	in situ gel, temperature responsive	50 μL	(1) no injections/implants; (2) baseline
Patel et al. (2022)^30^	neovascular retinal disease	axitinib	modified PEG; sodium phosphate	not specified	not specified	implant	700 μg (volume/exact size not specified)	(1) PBS/saline injection, no hydrogel; (2) baseline

### 2.2 Risk of Bias

The risk of bias for all included studies was evaluated using the SYRCLE risk-of-bias tool, which is specifically designed for animal studies and encompasses ten domains addressing selection, performance, detection, attrition, and reporting bias.^31^ Overall, the majority of studies were classified as having an “unclear” risk of bias across several domains (Table S3). This classification primarily reflected insufficient reporting of methodological details rather than evidence of systematic bias.

Specifically, many studies lacked explicit descriptions of randomization procedures, allocation concealment, housing conditions, blinding of investigators, and outcome assessment methods. Only 44.4% (4/9) of studies clearly reported whether samples were excluded from analysis and provided justification for exclusion. The use of investigator (1/9) and outcome assessor (2/9) blinding was rarely employed; however, blinding was often impractical given the intensive monitoring required for these experiments. None of the included studies explicitly described concealed allocation procedures, randomized housing, or randomized outcome selection.

Baseline animal characteristics were adequately reported in 55.6% (5/9) of studies, while the remaining studies omitted limited details, such as average body weight. With respect to reporting bias, all but one study demonstrated low risk, as outcome reporting was consistent with described experimental methods and sufficient detail was provided to support the reported results. Despite incomplete reporting across several SYRCLE domains, the procedural methodologies and outcome measurements were generally well described, and the experimental approaches were appropriate for the stated objectives. As such, the overall quality of the included studies supports the validity of the conclusions drawn, while highlighting the need for improved methodological transparency in future preclinical investigations.

### 2.3 Meta-Analysis

#### 2.3.1 IOP

Meta-analysis results for IOP changes relative to baseline measurements (obtained prior to treatment) are presented in Figure 2. Effect sizes are reported as standardized mean differences (SMDs) with corresponding 95% confidence intervals (CI), separated by post-treatment interval (14 days, 28–30 days, and 50–60 days).

**Figure 2:**
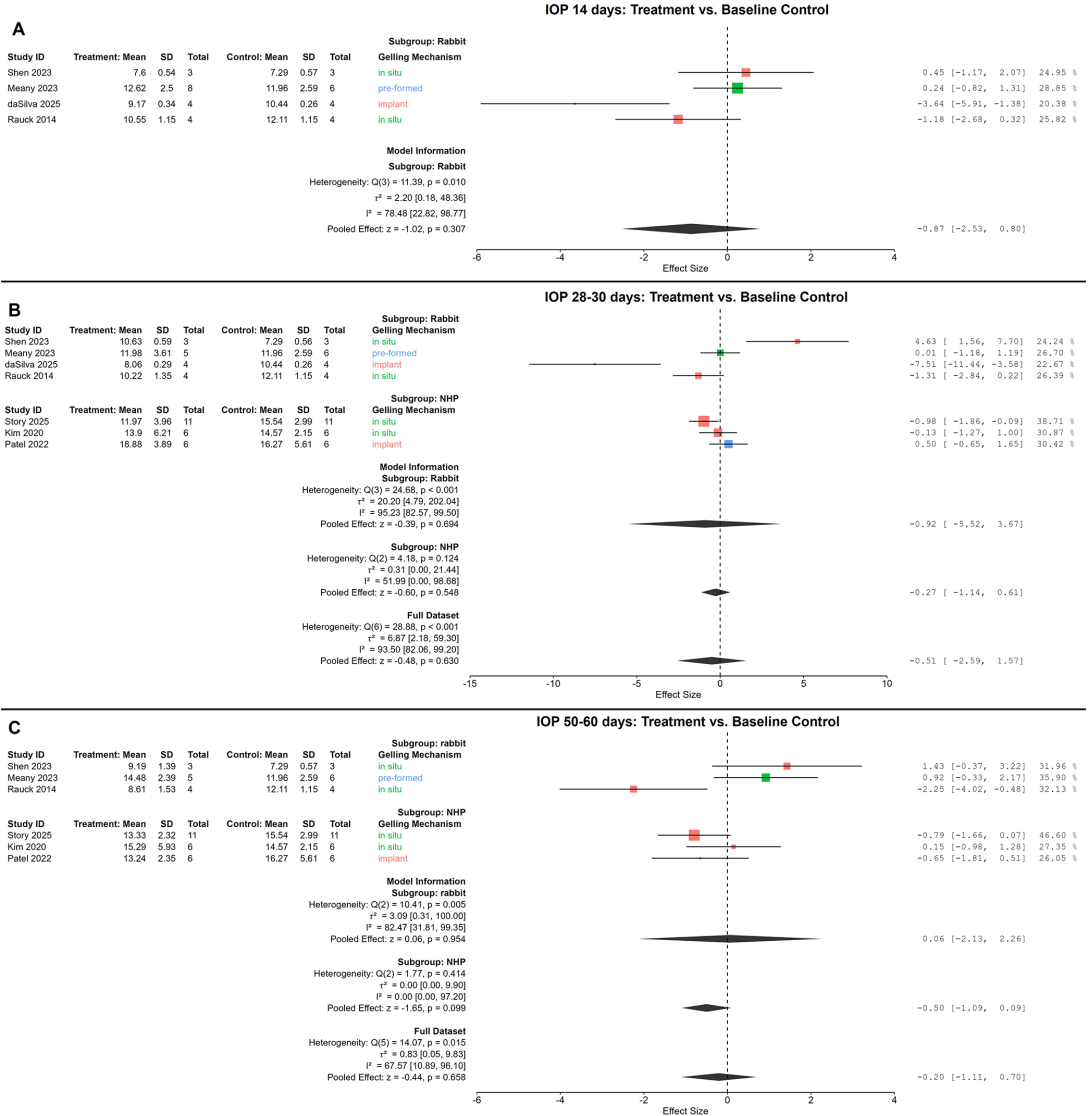
Meta-analysis of intraocular pressure changes comparing drug-loaded hydrogel–treated eyes to baseline measurements. Results are shown for (A) 14 days, (B) 28–30 days, and (C) 50–60 days post-treatment. Black diamonds represent the pooled standardized mean difference (SMD) estimates. Horizontal black lines indicate the 95% confidence intervals (CI) for individual studies. The dotted vertical line denotes no effect (SMD = 0). Raw data are shown in the left panel, with corresponding SMD values, 95% CIs, and study weights reported in the right panel.

At 14 days, all included studies utilized rabbit models, yielding a pooled estimate of SMD = −0.87 (95% CI: −2.53, 0.80) (Figure 2A). By 28–30 days and 50–60 days, three additional NHP studies were included, with pooled estimates of −0.51 (95% CI: −2.59, 1.57) and −0.20 (95% CI: −1.11, 0.70), respectively (Figure 2B, C). Across all time points, effect sizes were centered near zero with nonsignificant p-values (*p* > 0.05), indicating no clinically meaningful increase in IOP following hydrogel administration. On average, IOP values showed a slight, nonsignificant decrease relative to baseline. Heterogeneity for baseline comparisons was moderate to high (*I* ^2^ = 68–94%), with similar values observed within rabbit subgroups (*I* ^2^ = 78–95%) and lower heterogeneity among NHP studies (*I* ^2^ = 0–52%) (Figure 2, Table S4). This variability likely reflects the inherent limitations of baseline comparisons, which are susceptible to time-based and environmental influences unrelated to treatment effects. Therefore, comparisons between hydrogel-treated eyes and untreated controls were subsequently evaluated to provide a more robust assessment of IOP changes.

Comparisons between hydrogel-treated eyes and untreated control groups demonstrated similar trends (Figure 3). A statistically significant IOP reduction was observed at 14 days (SMD = −1.81; 95% CI: −2.67, −0.94; *p* < 0.001), driven primarily by data from Hu et al. (2024),^24^ where surgical incisions likely contributed to transient IOP reduction (Figure 3A). At 28–30 days, a small, nonsignificant increase was observed (SMD = 0.53; 95% CI: −0.70, 1.77; p = 0.397), while at 50–60 days IOP again trended slightly lower (SMD = −0.41; 95% CI: −1.00, 0.19; *p* = 0.182) (Figure 3B,C). Heterogeneity among treated-versus-control comparisons was generally low to moderate across pooled estimates and animal subgroups (Figure 3; Table S4). The lower heterogeneity observed in this analysis likely reflects the use of concurrent control groups, which minimizes variability introduced by time-dependent changes inherent to baseline comparisons. The only analysis demonstrating substantial heterogeneity was the pooled estimate at 28–30 days (*I* ^2^ = 69.3%). This variability can be attributed to inherent differences between animal models, including species-specific physiology and variations in treatment protocols, as heterogeneity was low within animal-specific subgroup analyses.

**Figure 3:**
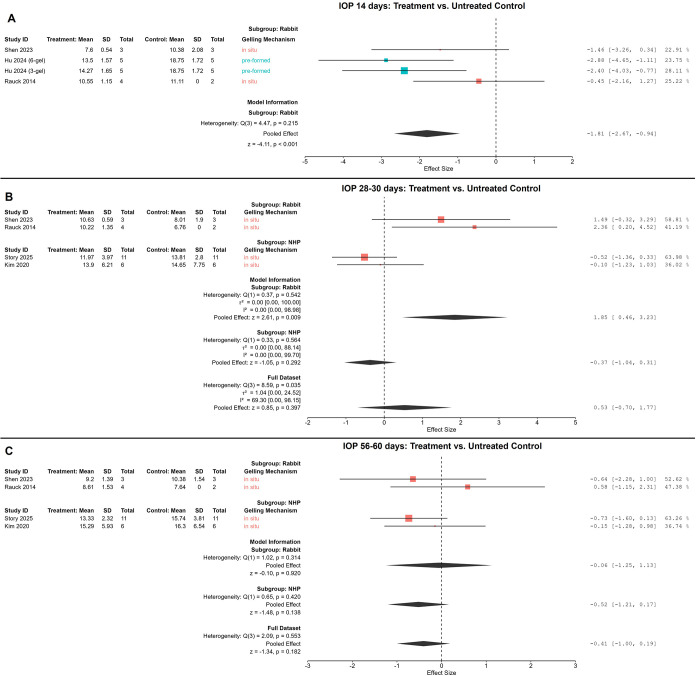
Meta-analysis of intraocular pressure changes comparing drug-loaded hydrogel–treated eyes to untreated controls. Results are shown for (A) 14 days, (B) 28–30 days, and (C) 56–60 days post-treatment. Black diamonds represent the pooled standardized mean difference (SMD) estimates. Horizontal black lines indicate the 95% confidence intervals (CI) for individual studies. The dotted vertical line denotes no effect (SMD = 0). Raw data are shown in the left panel, with corresponding SMD values, 95% CIs, and study weights reported in the right panel.

No significant differences were observed between rabbit and NHP subgroups, aside from the 28–30 day treatment-versus-untreated control comparison, where NHPs exhibited a lower SMD (−0.37; 95% CI: −1.04, 0.31) compared with rabbits (1.85; 95% CI: 0.46, 3.23) (Figure 3B, *p* = 0.005). Notably, this was the only time point at which either the pooled or subgroup analyses demonstrated a significant change in IOP, with a modest increase observed in rabbits (1.85; *p* = 0.009).

#### 2.3.2 Total Retinal Thickness

When comparing treatment to baseline at 14 days, the pooled SMD for TRT was 0.08 (95% CI: −0.76, 0.93) (Figure 4A). At 28–30 days and 50–60 days, pooled estimates were 0.27 (95% CI: −0.35, 0.90) and −0.16 (95% CI: −0.69, 0.37), respectively (Figure 4B,4C). Across all time points, effect sizes were centered near zero with confidence intervals crossing zero and nonsignificant p-values (*p* > 0.05).

**Figure 4:**
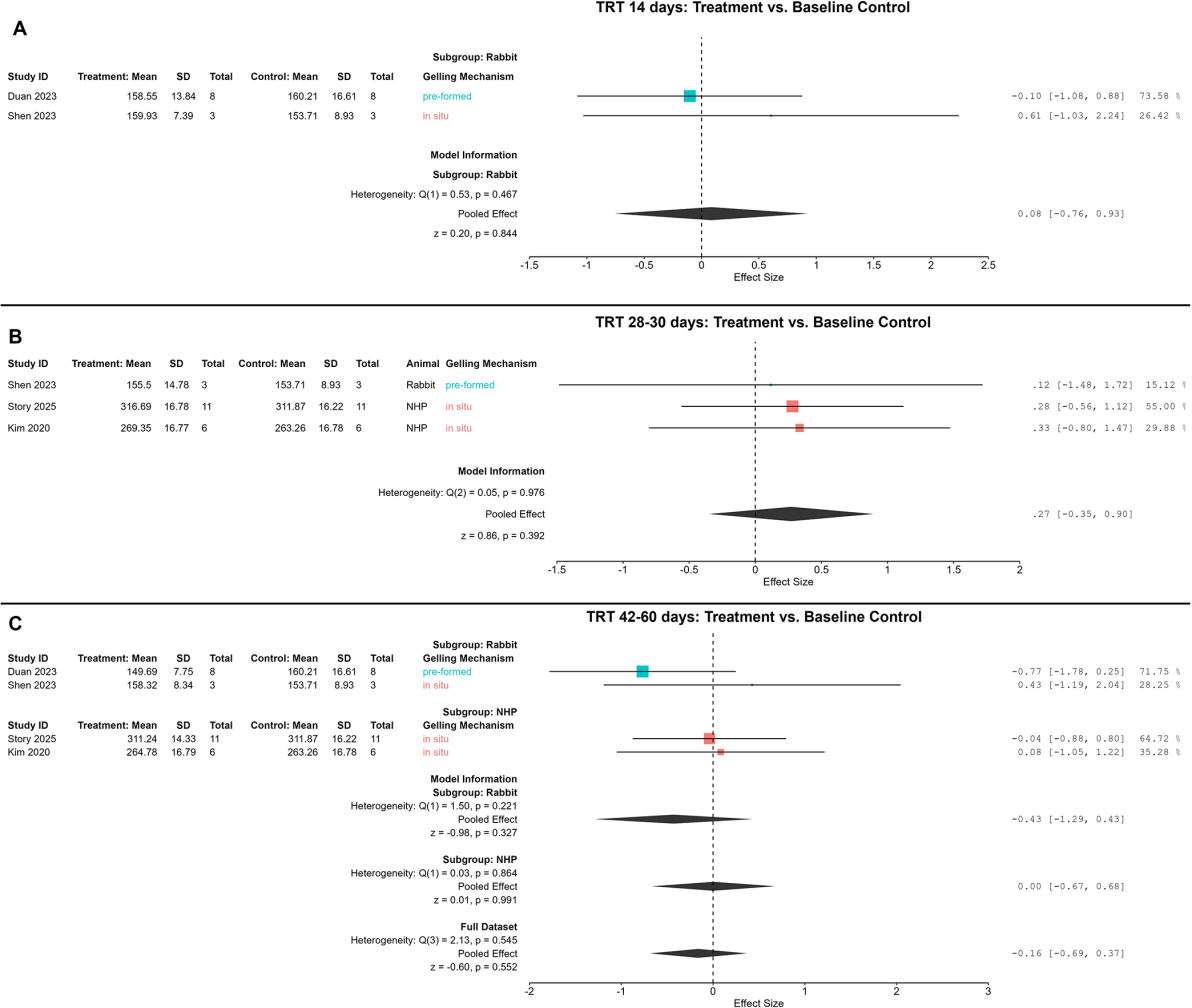
Meta-analysis of total retinal thickness changes comparing drug-loaded hydrogel–treated eyes to baseline measurements. Results are shown for (A) 14 days, (B) 28–30 days, and (C) 42–60 days post-treatment. Black diamonds represent the pooled standardized mean difference (SMD) estimates. Horizontal black lines indicate the 95% confidence intervals (CI) for individual studies. The dotted vertical line denotes no effect (SMD = 0). Raw data are shown in the left panel, with corresponding SMD values, 95% CIs, and study weights reported in the right panel.

Similarly, comparisons between hydrogel-treated eyes and untreated control groups revealed no statistically significant differences at any time point (Figure 5). SMDs of 0.11 (95% CI: −0.58, 0.80), 0.22 (95% CI: −0.35, 0.79), and 0.05 (95% CI: −0.68, 0.57) for 14 days, 28–30 days, and 56–60 days respectively indicated no clinically meaningful change in TRT following hydrogel administration. Heterogeneity across all TRT analyses, including pooled and subgroup comparisons, was low (*I* ^2^ = 0–33%; Table S4). Moreover, no significant differences in TRT were observed between rabbit and NHP models.

**Figure 5:**
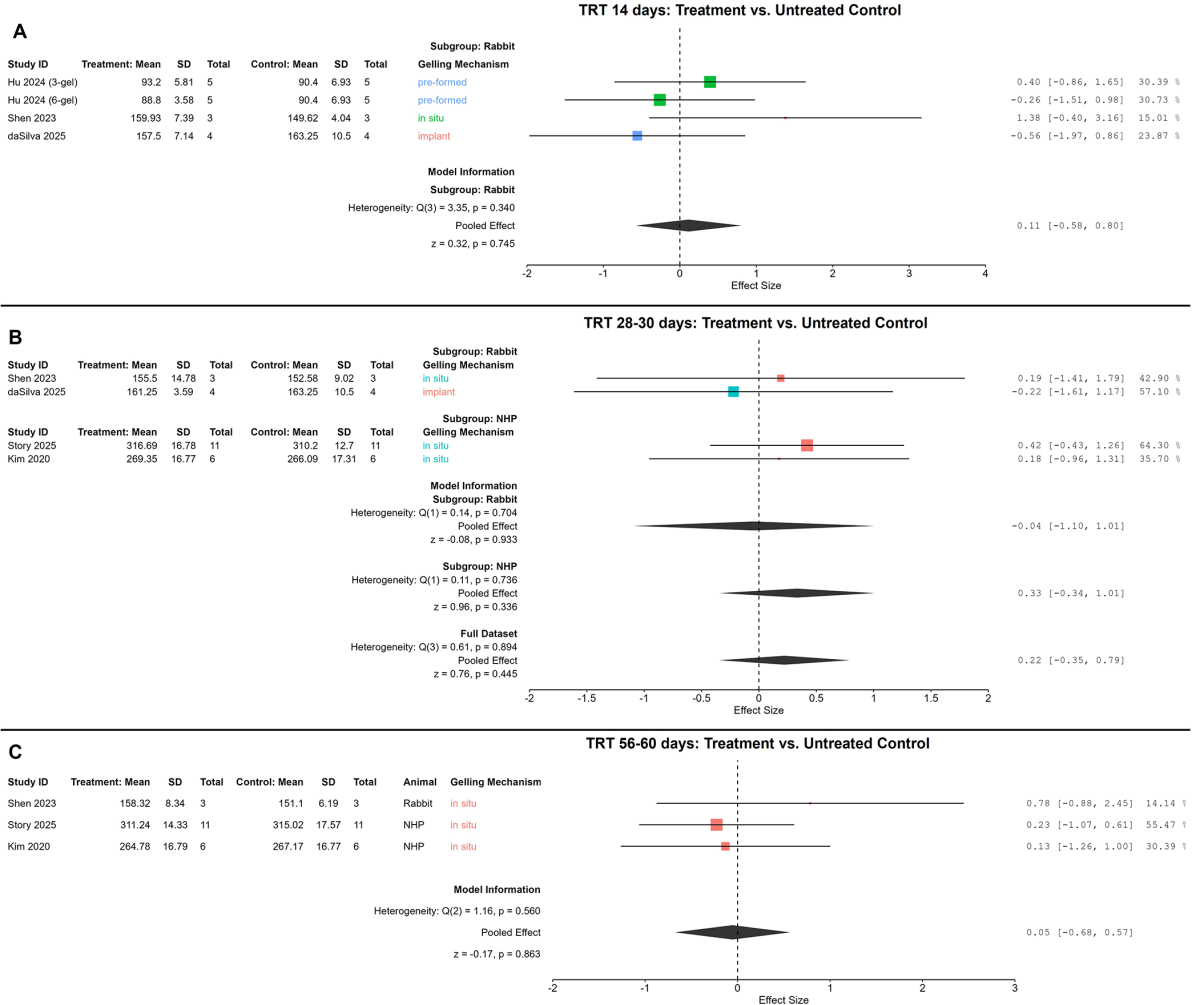
Meta-analysis of total retinal thickness changes comparing drug-loaded hydrogel–treated eyes to untreated controls. Results are shown for (A) 14 days, (B) 28–30 days, and (C) 56–60 days post-treatment. Black diamonds represent the pooled standardized mean difference (SMD) estimates. Horizontal black lines indicate the 95% confidence intervals (CI) for individual studies. The dotted vertical line denotes no effect (SMD = 0). Raw data are shown in the left panel, with corresponding SMD values, 95% CIs, and study weights reported in the right panel.

## 3 Discussion

Preclinical studies investigating intravitreal (IVT) hydrogels for drug delivery are extensive, encompassing a broad range of polymeric systems, therapeutic classes, and disease indications, including glaucoma, neovascular AMD, and retinal neovascularization.^19,32^ Given the clinical potential of such systems, data supporting their safety within the ocular space would be critical to successful translation of these products. While human data provides the greatest translational relevance, to date, only a single IVT hydrogel has advanced to clinical trials.^33^ Accordingly, the present study analyzed data from rabbit and nonhuman primate (NHP) models, as these species more closely recapitulate human ocular drug exposure profiles and pharmacokinetic parameters than rodent models.^34,35^

Meta-analytic outcomes focused on two clinically relevant end points, intraocular pressure (IOP) and total retinal thickness (TRT), to evaluate the short- to medium-term ocular safety of IVT hydrogel formulations. IOP reflects the balance between aqueous humor inflow and outflow, and deviations from physiological levels can compromise ocular perfusion and lead to vision-threatening conditions such as glaucoma, uveitis, and retinal detachment.^36,37^ Sudden elevations in IOP may induce mechanical stress and restrict retinal blood flow, while sustained increases are associated with retinal ganglion cell loss, optic nerve damage, and thinning of the retinal nerve fiber layer.^37^ Accordingly, IOP is a critical safety end point for intraocular drug delivery systems.

Standard IVT injections are known to cause transient IOP spikes, with sustained elevations reported in some patients, particularly following repeated administration.^38^ Corticosteroid therapies carry a higher risk of persistent IOP elevation than anti-VEGF agents, and injection frequency has been identified as a key risk factor for long-term IOP dysregulation.^39^ Moreover, retinal thickness serves as a sensitive indicator of retinal toxicity, as thinning can reflect early structural damage to retinal layers.^40^ Given the interplay between sustained IOP elevations and retinal structural integrity, concurrent assessment of IOP and TRT provides a robust evaluation of intraocular safety.

Across all analyzed data sets, no significant changes in IOP or TRT were observed from 14 days to two months postinjection, with the exception of a modest increase in IOP for the rabbit subgroup at 28–30 days. While standard IVT injections often cause transient pressure spikes due to acute volume displacement, as well as sustained pressure increases in some patients, the hydrogel formulations analyzed showed effect sizes centered near zero. Moreover, when assessing gelation mechanism (implant, preformed injectable, or in situ), no consistent differences in IOP or TRT were observed across the three modalities. The inclusion of diverse therapeutics, hydrogel chemistries, and animal models supports the generalizability of these findings across a broad range of IVT hydrogel systems.

The majority of meta-analytic evaluations demonstrated low heterogeneity (*I* ^2^ < 40%). Subgrouping by animal model effectively reduced heterogeneity in most instances, confirming that species-specific differences account for a portion of the observed variance. The high degree of consistency across the meta-analyzed data is noteworthy, given the inherent variability tied to polymer composition, therapeutic payload, animal age, and differences in injection or implantation procedures. This suggests that the results presented are robust, despite inherent differences across independent studies.

The only analysis that consistently demonstrated moderate to high heterogeneity was the IOP comparisons utilizing baseline control measurements. In most cases, baseline controls are not adequate to replace untreated control groups since they cannot control for changes that occur over time such as circadian rhythm, body temperature, or other physiological changes.^41^ This suggests that the observed heterogeneity was tied to physiological and/or experimental differences over time, and emphasizes that a untreated control group that can be measured in parallel with treated groups provides a more accurate control measure.

Despite these encouraging findings, several limitations should be acknowledged. First, the scope of this meta-analysis was restricted by the small number of studies that met the stringent inclusion criteria. Of the nine included studies, eight reported IOP data and five reported TRT data, highlighting the need for larger data sets as additional preclinical evidence becomes available. Risk-of-bias assessment using the SYRCLE tool identified predominantly unclear risk across most domains, largely due to insufficient reporting of randomization, blinding, and allocation concealment. In addition, all but one study utilized healthy animal models, which may not fully capture hydrogel behavior in diseased or aged eyes where vitreous structure, ocular fluid dynamics, and clearance mechanisms can be altered.^42–44^ Consequently, the safety profiles observed in the present analysis may not fully reflect outcomes in clinical populations.

The analysis was further limited by the follow-up durations reported in the literature. Few studies evaluated IOP or TRT during the immediate postinjection period (≤2 weeks), preventing assessment of transient physiological responses that may occur shortly after intravitreal administration. Additionally, the longest follow-up duration consistently reported across studies was approximately two months, limiting conclusions regarding long-term safety outcomes such as complete material degradation, the effects of degradation byproducts, chronic inflammatory responses, foreign-body reactions, and long-term clearance. Therefore, while the present findings support favorable short- to medium-term tolerability, they cannot fully establish the long-term safety profile of intravitreal hydrogel systems. Finally, although IOP and TRT represent clinically relevant indicators of ocular safety, these parameters alone do not fully characterize intraocular biocompatibility. Additional outcomes, including chronic inflammation, retinal structural changes, trabecular meshwork remodeling, and other subclinical responses, are necessary to provide a more comprehensive assessment of hydrogel safety.

To support successful clinical translation, future preclinical studies should adopt standardized reporting frameworks such as the ARRIVE guidelines to enhance transparency and reproducibility.^45,46^ Earlier, more frequent, and longer-duration post-treatment assessments, together with the inclusion of aged and disease-relevant animal models, would enhance the translational relevance of safety evaluations. Furthermore, incorporation of standardized histological assessments and molecular markers of inflammation would provide a more comprehensive evaluation of intraocular biocompatibility. As additional data sets become available, future analyses may further investigate the influence of therapeutic class, biomaterial composition, and disease state on ocular safety outcomes.

## 4 Materials and Methods

### 4.1 Search Strategy

A comprehensive literature search was conducted across PubMed, Web of Science, and Scopus in June of 2025, following PRISMA guidelines and guided by the PICO framework (Table 3). To maximize inclusivity, broad search terms were employed: (*intravitreal OR intravitreous OR vitreous injection*) *AND* (*hydrogel OR* in situ *gel OR 3D polymer network*). To ensure the search was comprehensive, additional search strategies in PubMed were performed on 10/09/2025 using combinations of (*hydrogel*) *AND* (*intravitreal*) *AND* (*rabbit OR nonhuman primate OR NHP*) *NOT vitreous substitute* to ensure all eligible studies were included.

**Table 3:** PICO Framework Used to Guide Systematic Review

PICO element	description
population	in vivo studies using rabbits or nonhuman primates
intervention	hydrogels administered by intravitreal injection/implantation designed for drug delivery applications
comparisons	no treatment or baseline measurement prior to treatment
outcome measures	changes in intraocular pressure (IOP) and/or total retinal thickness (TRT)

### 4.2 Eligibility Criteria

Original research articles or conference proceedings that reported quantitative values for IOP and/or TRT measurements after hydrogel injection were considered. Only hydrogels intended for drug-delivery applications that were intravitreally injected into the eye of rabbits or nonhuman primates (NHPs) were included. Studies investigating vitreous substitutes were excluded since vitrectomy procedures were outside of the scope of the current review.

### 4.3 Data Collection and Extraction

All search results were imported into Covidence systematic review software (Veritas Health Innovation, Melbourne, Australia),^47^ where duplicate articles were removed. Remaining papers went through two rounds of review: (1) relevance screened by title and abstract and (2) relevance screened by full text. In the first screen, papers were primarily excluded due to not being a primary article, including the wrong route of administration, being a vitreous substitute, or not including a hydrogel. In the second screen, articles were primarily excluded for not using an appropriate animal model or not including necessary outcome parameters.

Relevant characteristics of included studies were recorded, including the hydrogel components used, therapeutic used, disease targeted, animal strain employed, volume and method for injection/implantation, experimental methods for IOP and TRT assessments, treatment group details, sample size, and methods of data reporting. The reported IOP and TRT measurements were extracted for all reported time points. When numerical values were not directly available as a table format, they were extracted from published graphs using GetData Graph Digitizer (Informer Technologies Inc., Los Angeles, CA). Time points assessed spanned from 2 weeks to 6 months postinjection, based on the availability of comparable time points reported across individual studies.

Retinal thickness was assessed using TRT values as reported in each study. In one study (Hu et al., 2024),^24^ TRT was not directly reported and was therefore calculated by summing the five individual retinal layer thicknesses provided. When variability was reported as the standard error of the mean (SEM), values were converted to standard deviation (SD) prior to effect-size calculation using the equation:

$$\mathrm{SD}=\mathrm{SEM}\times \sqrt{n}$$

### 4.4 Risk of Bias

Risk of bias was assessed for each study using the SYRCLE Risk of Bias (ROB) tool, which includes 10 items across 6 domains.^31^ Each item was independently evaluated by two reviewers (A.K.B.-W. and B.B.) and classified as “Yes,” “No,” or “Unclear.” Discrepancies were resolved through discussion to reach consensus (Table S3).

### 4.5 Data Analysis

The primary outcome was a change in IOP and TRT between the hydrogel injection group and control groups. Relevant comparisons were considered as the drug-loaded hydrogel compared to available controls including: (1) no treatment or (2) baseline measurements taken prior to hydrogel injection/implantation.

Standardized mean differences (SMDs) and corresponding standard errors were calculated from reported mean IOP or TRT values, standard deviations, and sample sizes. The Residual Heterogeneity test was used to assess significant heterogeneity in each analysis. This test was further supported by the *I* ^2^ statistic where <40%, 30–60%, and >60% was interpreted as low, moderate, and substantial heterogeneity, respectively.^48^ A random-effects model (Restricted ML) was used when moderate to substantial heterogeneity was observed, while fixed-effect models were used when heterogeneity was low.^49^ All analyses were performed using JASP (Version 0.95.11.0; JASP Team, Amsterdam, The Netherlands) with the Metafor R package.^50,51^ Statistical significance was defined as *p* ≤ 0.05.

Subgroup analyses were performed based on animal species (rabbit or NHP) to account for species-specific effects, with independent models fitted for each subgroup when sufficient data were available. When fewer than two data sets were available for a given species, analyses were conducted using the pooled data set without subgroup stratification. The mechanism of gel formation (in situ, preformed injectable, or implant) was recorded for each study and annotated in the analyses. Separate meta-analyses were performed for each control comparison at time points common across studies (14 days, 28–30 days, and 50–60 days post-treatment).

## 5 Conclusion

This meta-analysis demonstrates that intravitreally administered hydrogels maintain a favorable intraocular safety profile across a range of preclinical models and polymer biomaterials. By synthesizing data from rabbit and nonhuman primate studies, the analysis found no evidence of clinically meaningful elevations in IOP or adverse changes in TRT between 14 days and two months following administration. The stability of IOP and TRT measurements suggests that hydrogels, regardless of gelling mechanism or polymer composition, do not obstruct aqueous humor outflow or induce substantial mechanical stress within the vitreous cavity during the observation period. Collectively, these findings provide quantitative evidence supporting the short- to medium-term intraocular tolerability of drug-loaded hydrogels and their potential as biocompatible alternatives to the frequent intravitreal injections currently required for chronic retinal disease management. Future studies should prioritize extended follow-up durations to establish the long-term safety profile of intraocular hydrogel systems.

## Supplementary Material



## Notes

Data supporting this article have been included as part of the Supporting Information; risk of bias assessment; raw data for meta-analysis; and heterogeneity assessment (PDF)

## Abbreviations

HA: hyaluronic acid
ADH: adipic acid dihydrazide
PF127: pluronic 127
PBS: phosphate buffered saline
CHO: aldehyde terminated
GelMA: gelatin methacryloyl
LAP: lithium phenyl-2,4,6- trimethybenzoylphosphinate
HPMC: hypromellose
NP: nanoparticles
PEG: polyethylene glycol
PLA: polylactic acid
PLGA: poly­(lactic-*co*-glycolic acid)
PSHU: poly­(ethylene glycol)-poly­(serinol hexamethylene urethane)
PLLA: poly­(ethylene glycol)-*co*-(L-lactic acid)
NIPAAm: *N*-isopropylacrylamide
TEMED: tetramethylethylenediamine
DA: diacrylate
IOP: intraocular pressure
TRT: total retinal thickness
IVT: intravitreal
NHP: nonhuman primate
PRISMA: Preferred Reporting Items for Systematic reviews and Meta-Analyses
PICO: Population, Intervention, Comparison, Outcome
SYRCLE: Systematic Review Centre for Laboratory animal Experimentation
