# Supplementary material for: Safety of Long-Acting Intravitreal Hydrogels: A Comprehensive Evaluation of Intraocular Pressure and Retinal Integrity across Rabbit and Non-Human Primate Models
Source: ACS Pharmacol Transl Sci. 2026 Jul 12;9(8):2105–16. doi: 10.1021/acsptsci.6c00243 (PMC13482885; doi:10.1021/acsptsci.6c00243)
Supplement: Supplementary file 1 [file pt6c00243_si_001.pdf]

Supporting Information

Safety of Long-Acting Intravitreal Hydrogels: A Comprehensive Evaluation of Intraocular Pressure and Retinal Integrity Across Rabbit and Non-Human Primate Models

Alexa K. Beathard-Wojan[a], Becci Boughner[b], and Vibhuti Agrahari [a*]

[a] *Department of Pharmaceutical Sciences, University of Oklahoma Health Campus,*

1110 N Stonewall Ave, Oklahoma City, OK 73117, United States

[b] *Oklahoma School of Science and Mathematics,*

1141 N Lincoln Blvd Oklahoma City, OK 73104, United States

*Corresponding Author: Vibhuti-Agrahari@ou.edu

ORCID: Alexa K. Beathard-Wojan: 0009-0001-5213-1542

ORCID: Vibhuti Agrahari: 0000-0003-1884-1644

Table of Contents

Table S1. Raw IOP data used for meta-analytic assessments…..……………………………………………S3-S4

Table S2. Raw TRT data used for meta-analytic assessments.…..………………………………………...S4-S5

Table S3. Risk of Bias Assessment Results……………………………………………………………..S6

Table S4. Heterogeneity Assessment Results.….…………………………………………………………..S7

Table S1: Raw IOP data used for meta-analytic assessments.								
	Drug loaded hydrogel vs no treatment: 2 weeks							
Study ID	Average IOP (treated)	STDEV (treated)	Number of Eyeballs (treated)	Average IOP (control)	STDEV (control)	Number of Eyeballs (control)	Animal	Gelling Mechanism
Shen 2023	7.60	0.54	3	10.38	2.08	3	Rabbit	in situ
Hu 2024 (6-gel)	13.5	1.57	5	18.75	1.72	5	Rabbit	pre-formed
Hu 2024 (3-gel)	14.27	1.65	5	18.75	1.72	5	Rabbit	pre-formed
Rauck 2014	10.55	1.15	4	11.11	0	2	Rabbit	in situ
	Drug loaded hydrogel vs no treatment: 4 weeks-1 month							
Study ID	Average IOP (treated)	STDEV (treated)	Number of Eyeballs (treated)	Average IOP (control)	STDEV (control)	Number of Eyeballs (control)	Animal	Gelling Mechanism
Shen 2023	10.6336	0.59	3	8.01	1.90	3	Rabbit	in situ
Rauck 2014	10.22	1.35	4	6.76	0	2	Rabbit	in situ
Story 2025	11.97	3.96	11	13.81	2.80	11	NHP	in situ
Kim 2020	13.90	6.21	6	14.65	7.75	6	NHP	in situ
	Drug loaded hydrogel vs no treatment: 8 weeks-2 months							
Study ID	Average IOP (treated)	STDEV (treated)	Number of Eyeballs (treated)	Average IOP (control)	STDEV (control)	Number of Eyeballs (control)	Animal	Gelling Mechanism
Shen 2023	9.20	1.39	3	10.38	1.54	3	Rabbit	in situ
Rauck 2014	8.61	1.53	4	7.64	0	2	Rabbit	in situ
Story 2025	13.33	2.32	11	15.74	3.81	11	NHP	in situ
Kim 2020	15.29	5.93	6	16.30	6.54	6	NHP	in situ
	Drug loaded hydrogel vs baseline: 2 weeks							
Study ID	Average IOP (treated)	STDEV (treated)	Number of Eyeballs (treated)	Average IOP (control)	STDEV (control)	Number of Eyeballs (control)	Animal	Gelling Mechanism
Shen 2023	7.60	0.54	3	7.29	0.57	3	Rabbit	in situ
Meany 2023	12.62	2.50	8	11.96	2.59	6	Rabbit	pre-formed
daSilva 2025	9.17	0.34	4	10.44	0.26	4	Rabbit	implant
Rauck 2014	10.55	1.15	4	12.10	1.15	4	Rabbit	in situ
	Drug loaded hydrogel vs baseline: 4 weeks-1 month							
	Average IOP (treated)	STDEV (treated)	Number of Eyeballs (treated)	Average IOP (control)	STDEV (control)	Number of Eyeballs (control)	Animal	Gelling Mechanism

Shen 2023	10.63	0.59	3	7.29452	0.57	3	Rabbit	in situ
Meany 2023	11.98	3.61	5	11.96	2.59	6	Rabbit	pre-formed
daSilva 2025	8.06	0.29	4	10.44	0.26	4	Rabbit	implant
Rauck 2014	10.22	1.35	4	12.11	1.15	4	Rabbit	in situ
Story 2025	11.97	3.96	11	15.54	2.99	11	NHP	in situ
Kim 2020	13.90	6.21	6	14.57	2.15	6	NHP	in situ
Patel 2022	18.88	3.89	6	16.27	5.61	6	NHP	implant
	Drug loaded hydrogel vs baseline: 50-60 days							
Study ID	Average IOP (treated)	STDEV (treated)	Number of Eyeballs (treated)	Average IOP (control)	STDEV (control)	Number of Eyeballs (control)	Animal	Gelling Mechanism
Shen 2023	9.20	1.39	3	7.29	0.57	3	rabbit	in situ
Meany 2023	14.48	2.39	5	11.96	2.59	6	rabbit	pre-formed
Rauck 2014	8.61	1.53	4	12.11	1.15	4	rabbit	in situ
Story 2025	13.33	2.32	11	15.54	2.99	11	NHP	in situ
Kim 2020	15.29	5.93	6	14.57	2.15	6	NHP	in situ
Patel 2022	13.24	2.35	6	16.27	5.61	6	NHP	implant

Table S2: Raw TRT data used for meta-analytic assessments.								
	Drug loaded hydrogel vs no treatment: 2 weeks							
	Average IOP (treated)	STDEV (treated)	Number of Eyeballs (treated)	Average IOP (control)	STDEV (control)	Number of Eyeballs (control)	Animal	Gelling Mechanism
Hu 2024 (3-gel)	93.2	5.81	5	90.4	6.93	5	Rabbit	pre-formed
Hu 2024 (6-gel)	88.8	3.578	5	90.4	6.93	5	Rabbit	pre-formed
Shen 2023	159.93	7.388	3	149.62	4.04	3	Rabbit	in situ
daSilva 2025	157.5	7.14	4	163.25	10.5	4	Rabbit	implant
	Drug loaded hydrogel vs no treatment: 4 weeks-1 month							
	Average IOP (treated)	STDEV (treated)	Number of Eyeballs (treated)	Average IOP (control)	STDEV (control)	Number of Eyeballs (control)	Animal	Gelling Mechanism
Shen 2023	155.50	14.78	3	152.58	9.02	3	Rabbit	in situ
daSilva 2025	161.25	3.59	4	163.25	10.5	4	Rabbit	implant

Story 2025	316.69	16.78	11	310.20	12.70	11	NHP	in situ
Kim 2020	269.35	16.77	6	266.09	17.31	6	NHP	in situ
	Drug loaded hydrogel vs no treatment: 8 weeks-2 months							
	Average IOP (treated)	STDEV (treated)	Number of Eyeballs (treated)	Average IOP (control)	STDEV (control)	Number of Eyeballs (control)	Animal	Gelling Mechanism
Shen 2023	158.32	8.34	3	151.1	6.19	3	Rabbit	in situ
Story 2025	311.24	14.33	11	315.02	17.57	11	NHP	in situ
Kim 2020	264.78	16.79	6	267.17	16.77	6	NHP	in situ
	Drug loaded hydrogel vs baseline: 2 weeks							
	Average IOP (treated)	STDEV (treated)	Number of Eyeballs (treated)	Average IOP (control)	STDEV (control)	Number of Eyeballs (control)	Animal	Gelling Mechanism
Duan 2023	158.55	13.84	8	160.21	16.61	8	Rabbit	pre-formed
Shen 2023	159.93	7.39	3	153.71	8.94	3	Rabbit	in situ
	Drug loaded hydrogel vs baseline: 4 weeks-1month							
	Average IOP (treated)	STDEV (treated)	Number of Eyeballs (treated)	Average IOP (control)	STDEV (control)	Number of Eyeballs (control)	Animal	Gelling Mechanism
Shen 2023	155.50	14.78	3	153.71	8.94	3	Rabbit	pre-formed
Story 2025	316.69	16.78	11	311.87	16.22	11	NHP	in situ
Kim 2020	269.35	16.77	6	263.26	16.78	6	NHP	in situ
	Drug loaded hydrogel vs baseline: 6weeks-2month							
	Average IOP (treated)	STDEV (treated)	Number of Eyeballs (treated)	Average IOP (control)	STDEV (control)	Number of Eyeballs (control)	Animal	Gelling Mechanism
Duan 2023	149.69	7.76	8	160.208	16.61	8	Rabbit	pre-formed
Shen 2023	158.32	8.34	3	153.711	8.94	3	Rabbit	in situ
Story 2025	311.24	14.33	11	311.865	16.22	11	NHP	in situ
Kim 2020	264.78	16.79	6	263.261	16.78	6	NHP	in situ

Table S3: Risk of Bias Assessment Results.										
Study ID	**Sequence Generation:** Was the allocation sequence adequately generated and applied?	**Baseline Characteristics:** Were the groups similar at baseline or were they adjusted for confounders in the analysis?	**Allocation Concealment:** Was the allocation adequately concealed?	**Random Housing:** Were the animals randomly housed during the experiment?	**Blinding:** Were the caregivers and/or investigators blinded from knowledge which intervention each animal received during the experiment?	**Random Outcome Assessment:** Were animals selected at random for outcome assessment?	**Blinding:** Was the outcome assessor blinded?	**Incomplete Outcome Data:** Were incomplete outcome data adequately addressed?	**Selective Outcome Reporting:** Are reports of the study free of selective outcome reporting?	**Other:** Was the study apparently free of other problems that could result in high risk of bias?
Story 2025	unclear	yes	unclear	unclear	unclear	unclear	unclear	yes	yes	yes
Duan 2023	yes	yes	unclear	unclear	unclear	unclear	yes	yes	yes	yes
Shen 2023	unclear	unclear	unclear	unclear	unclear	unclear	unclear	unclear	yes	yes
Kim 2020	unclear	yes	unclear	unclear	unclear	unclear	unclear	no	yes	yes
Hu 2024	unclear	yes	unclear	unclear	yes	unclear	yes	unclear	yes	yes
Meany 2023	unclear	unclear	unclear	unclear	unclear	unclear	unclear	unclear	yes	yes
daSilva 2025	yes	yes	unclear	unclear	no	unclear	no	yes	yes	yes
Patel 2022	unclear	unclear	unclear	unclear	unclear	unclear	unclear	unclear	unclear	yes
Rauck 2014	unclear	unclear	unclear	unclear	unclear	unclear	unclear	unclear	yes	yes

Table S4: Heterogeneity Assessment Results						
	2-week		28-30 days		50-60 days	
	I^2	P-value	I^2	P-value	I^2	P-value
	IOP - Baseline Control					
Rabbit	78.477	0.01	95.234	< .001	82.469	0.005
NHP	n/a	n/a	51.994	0.124	0	0.414
Pooled	78.477	0.01	93.501	< .001	67.566	0.015
	IOP - Untreated Control					
Rabbit	32.926	0.215	0	0.542	1.562	0.314
NHP	n/a	n/a	0	0.564	0	0.42
Pooled	32.926	0.215	69.299	0.035	0	0.553
	Retinal Thickness - Baseline Control					
Rabbit	0	0.467	n/a	n/a	33.24	0.221
NHP	n/a	n/a	n/a	n/a	0	0.864
Pooled	0	0.467	0	0.976	0	0.545
	Retinal Thickness - Untreated Control					
Rabbit	0.001	0.34	0	0.704	n/a	n/a
NHP	n/a	n/a	0	0.736	n/a	n/a
Pooled	0.001	0.34	0	0.894	0	0.56

*Green boxes indicate data analyzed using a random-effects model. All other data analyzed using fixed effect model.
